# Clinical Evidence for Q10 Coenzyme Supplementation in Heart Failure: From Energetics to Functional Improvement

**DOI:** 10.3390/jcm9051266

**Published:** 2020-04-27

**Authors:** Anna Di Lorenzo, Gabriella Iannuzzo, Alessandro Parlato, Gianluigi Cuomo, Crescenzo Testa, Marta Coppola, Giuseppe D’Ambrosio, Domenico Alessandro Oliviero, Silvia Sarullo, Giuseppe Vitale, Cinzia Nugara, Filippo M. Sarullo, Francesco Giallauria

**Affiliations:** 1 Department of Translational Medical Sciences, “Federico II” University of Naples, 80131 Naples, Italy; dilorenzoanna2@gmail.com (A.D.L.); alessandroparlato96@gmail.com (A.P.); gianluigi.cuomo95@gmail.com (G.C.); kre.testa@gmail.com (C.T.); marta.coppola@live.it (M.C.); giuseppe.dambrosio91@gmail.com (G.D.); alessandro.oliviero0695@gmail.com (D.A.O.); 2 Department of Clinical Medicine and Surgery, “Federico II” University of Naples, 80131 Naples, Italy; gabriella.iannuzzo@unina.it; 3 Cardiovascular Rehabilitation Unit, Buccheri La Ferla Fatebenefratelli Hospital, 90123 Palermo, Italy; silvia.sarullo@gmail.com (S.S.); giuseppevit@hotmail.com (G.V.); cinzianugara@gmail.com (C.N.); sarullo.filippo@fbfpa.it (F.M.S.)

**Keywords:** heart failure, coenzyme Q10, coenzyme Q10 deficiency, coenzyme Q10 supplementation, heart failure mortality

## Abstract

Oxidative stress and mitochondrial dysfunction are hallmarks of heart failure (HF). Coenzyme Q10 (CoQ10) is a vitamin-like organic compound widely expressed in humans as ubiquinol (reduced form) and ubiquinone (oxidized form). CoQ10 plays a key role in electron transport in oxidative phosphorylation of mitochondria. CoQ10 acts as a potent antioxidant, membrane stabilizer and cofactor in the production of adenosine triphosphate by oxidative phosphorylation, inhibiting the oxidation of proteins and DNA. Patients with HF showed CoQ10 deficiency; therefore, a number of clinical trials investigating the effects of CoQ10 supplementation in HF have been conducted. CoQ10 supplementation may confer potential prognostic advantages in HF patients with no adverse hemodynamic profile or safety issues. The latest evidence on the clinical effects of CoQ10 supplementation in HF was reviewed.

## 1. Introduction

**Figure 1:**
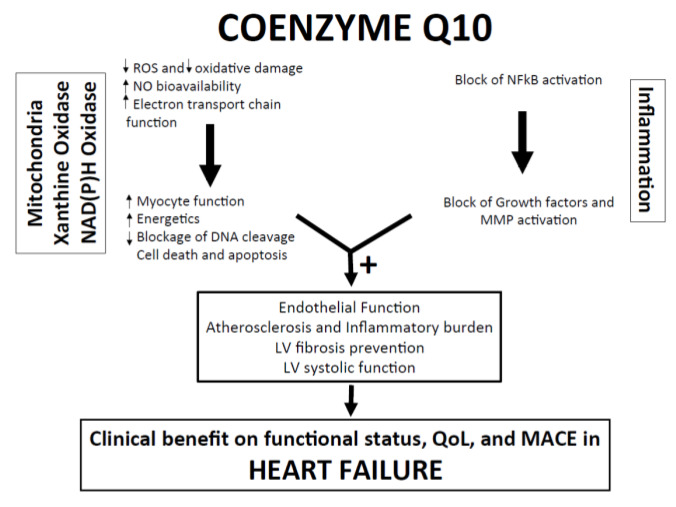
Pathophysiological mechanisms and potential clinical benefits of CoQ10 in heart failure. LV Left: Ventricle; MACE: Major Adverse Cardiovascular Events; MMP: Matrix Metalloproteinase; NAD(P)H: Nicotinamide adenine dinucleotide phosphate; NFkB: nuclear factor kappa-light-chain-enhancer of activated B cells; NO: Nitric Oxyde; QoL: Quality of Life; ROS: Reactive Oxygen Species.

Despite brilliant research development in medical and device therapies, heart failure (HF) still remains a complex multifaceted syndrome with poor outcomes [1,2,3]. Mitochondrial dysfunction is a hallmark of HF syndrome, predominantly characterized by a deficit in the production of myocardial adenosine triphosphate, derailment of calcium exchange and increased production of reactive oxygen species leading to endothelial dysfunction [4,5,6] (Figure 1).

Current therapies for chronic HF treatment act by modulating derailed neurohormonal pathways (i.e., renin–angiotensin–aldosterone system) or by inhibiting the neprilysin pathway [7,8]. These therapeutic strategies are usually limited by side effects limiting up-titration of doses; electrolyte disorders and hypotension are the main side effects; therefore the ideal drug should have hemodynamic neutral profiles [9,10]. In addition, in patients with chronic HF, exercise training has proven to exert beneficial effects on exercise tolerance and functional capacity, cardiac remodeling, morbidity and mortality and quality of life [7,8,11,12,13,14,15,16,17,18,19,20,21,22,23,24,25,26,27,28,29].

Since Coenzyme Q10 (CoQ10) plays a key role in cell energetics acting as an effective anti-inflammatory agent exerting endothelial function improvement, it may represent a plausible therapeutic option for HF patients [30,31,32,33,34]. Interestingly, lower CoQ10 levels have been found in more compromised HF patients presenting with high New York Heart Association (NYHA) class and reduced left ventricular ejection fraction (HFrEF). CoQ10 supplementation may potentially confer prognostic advantages in HFrEF [35] neutrally impacting on hemodynamic profile and without safety issues [36].

The present review summarizes the latest evidence of the clinical effects of CoQ10 supplementation in HF.

## 2. Mitochondria Dysfunction and Energy Depletion in HF

Chronic HF inexorably progresses intermittently, with relatively steady phases alternated with acute decompensation needing therapy upgrading or hospitalization. Furthermore, although conventional drugs may ameliorate morbidity and mortality rates, specific HF symptoms (i.e., fatigue and exercise intolerance) remain a major challenge for physicians [37].

Modulating cardiac energetics might represent an intriguing therapeutic option [38,39]. It has been postulated that energy depletion is the main constant of the failing and decompensated heart, which requires more energy to maintain homeostasis [40,41]. Abnormal calcium handling, ATP depletion and mitochondrial dysfunction derailing cardiac metabolic pathways are all common findings in HF patients [39,40,41]. These alterations lead to energy depletion that negatively affects cardiac contractile function. Therapies counteracting cardiac energy exhaustion may play a role in HF management increasing the duration of compensation phases [38].

Mitochondrial dysfunction, led by activation of immune-inflammatory pathways and overproduction of reactive oxygen species (ROS), overwhelms the antioxidant cell enzyme defense and is associated with the initiation and progression of atherosclerosis [42]. Therefore, new approaches to support standard therapies of atherosclerosis are needed. CoQ10 has been shown to enhance ATP production as a carrier in the mitochondrial respiratory chain; furthermore, CoQ10 can improve endothelial function and mediate epigenetic regulation in genes involved in cell signaling [43].

## 3. CoQ10 Levels in Healthy Individuals and in Patients with Heart Failure

Serum CoQ10 level is approximately 1 µmol/L [44,45]. Since CoQ10 binds to lipoproteins, indexing CoQ10 to LDL (0.33 ± 0.01 μmol/L) [46] or total cholesterol (from 0.16 ± 0.05 to 0.24 ± 0.27 μmol/L) have been reported [47,48].

In patients with HF, CoQ10 levels are inversely associated with functional status and with a severity of HF symptoms such as fatigue, exercise tolerance and dyspnea. In a sample of 43 HF patients with heterogeneous etiology, endomyocardial biopsies showed that myocardial CoQ10 levels are inversely related to NYHA functional class: higher CoQ10 levels were observed in less compromised patients (NYHA class I and II patients); conversely, more compromised HF patients (NYHA class III and IV) had significantly lower myocardial CoQ10 levels [49]. CoQ10 supplementation efficiently restored CoQ10 levels both in the myocardium and sera [49]. The associations between CoQ10 levels and HF symptoms have been observed in other cohorts [35,50]. In the CORONA (Controlled Rosuvastatin Multinational Study in HF) trial (*n* = 1191), the lowest CoQ10 levels were significantly associated either to the lowest left ventricular ejection fraction (LVEF) or to the highest natriuretic peptide levels [51].

## 4. Clinical Data on CoQ10 Supplementation in HF

Several trials investigated the effects of CoQ10 supplementation in HF; the majority of them predominantly report data on HFrEF [52,53,54,55,56,57,58,59,60,61,62], only one trial was designed for patients with HF with preserved ejection fraction (HFpEF) [63] (Table 1). Principal data from the meta-analysis are reported in Table 2.

### 4.1. Effects of CoQ10 Supplementation on Functional Status

Significant effects of CoQ10 supplementation in improving functional status as evaluated by NYHA class have been reported in several trials [52,53,54,55,56,57,58] (Table 1). In a randomized double-blind placebo-controlled trial enrolling 39 HF patients, Keogh et al. showed a significant reduction of NYHA class (from 2.9 ± 0.06 to 2.4 ± 0.01; *p* = 0.001) in the supplementing therapy group (CoQ10 50 mg 3/d for three months) but no significant differences in the placebo group (*p* = 0.01 between groups) [52]. Berman et al. [53] reported NYHA class improvement (NYHA class from 3.1 to 2.4; *p* = 0.01) in 32 HF patients supplementing CoQ10 (60 mg 2/d for three months) vs. placebo (*p* = 0.01). Moreover, in 62 patients with HFrEF of mixed pathogeneses, four-month CoQ10 supplementation showed significant improvement of the NYHA class (from 2.7 ± 0.7 to 2.3 ± 0.7, *p* = 0.025) vs. placebo (2.9 ± 0.8 to 2.7 ± 0.7, *p* = 0.17; between-group *p* = 0.002) [54] (Table 1).

In the Q10 SYMptoms, Biomarker status, and long-term Outcome (Q-SYMBIO) multicenter trial, Mortensen et al. [55] found significant results in long-term CoQ10 administration (two-years of supplementing CoQ10 100 mg 3/d). Improvement for at least one NYHA class grade from baseline has been reached in 86 HF patients compared to placebo (58% vs. 45%, *p* = 0.028) [55] (Table 1).

Finally, Soongswang et al. [58] showed significant improvement in the NYHA class in 15 pediatric patients (median age 4.4 years) with idiopathic chronic dilated cardiomyopathy (DCM) undergoing CoQ10 supplementation (3.1 ± 0.6 mg/kg/d for nine months, four patients had improved by one functional class and one patient had improved by two classes at nine months with CoQ10, *p* = 0.005, Table 1).

### 4.2. Effects of CoQ10 Supplementation on Echocardiographic Parameters

In 79 stable HFrEF patients, Hofman-Bang et al. [59] reported that patients undergoing CoQ10 supplement (50 mg orally TID vs. placebo for three months) showed a slight improvement in LVEF (Table 1). In 22 patients with HFrEF, Munkholm et al. [60] observed that CoQ10 supplementation (100 mg orally twice a day vs. placebo for one year) exerted a significant improvement in echo parameters of great clinical relevance (i.e., an increase in stroke index, mean pulmonary artery pressure and a reduction in pulmonary capillary wedge pressure); however, no significant changes were observed in the placebo group (Table 1). No effects were reported on LVEF [60] (Table 1). Similarly, CoQ10 supplementation (200 mg orally daily vs. placebo for six months) exerted neutral results on LVEF in 55 patients with HFrEF [61] (Table 1).

Conversely, in 62 HFrEF patients supplemented with CoQ10 (100 mg bid plus atorvastatin 10 mg/d orally for four months) vs. placebo, Pourmoghaddas et al. [54] reported significantly improved LVEF in the CoQ10 group (from 18.7% ± 10.3% to 24.2% ± 14.5%, *p* = 0.003) compared to placebo (from 26.2% ± 9.1% to 25.8% ± 9.7%, *p* = 0.23; between-group *p* = 0.006, Table 1). These findings were consistent with a more recent study by Mortensen et al. [61] confirming a significant increase in LVEF (+6%) in HFrEF patients treated with CoQ10 at 300 mg daily in addition to conventional therapy for two years compared to placebo (Table 1). Finally, two randomized trials reported no significant improvement in fractional shortening [62,63] (Table 1).

A significant improvement in LVEF was reported in a meta-analysis of 10 studies including 340 patients with HF (CoQ10 supplementation: 100 mg to 450 mg daily for a period from four weeks to two years, average six months) [77]. These findings were successively confirmed in a meta-analysis including 16 trials (*n* = 1662 HF patients supplemented with CoQ10 from 100 to 200 mg daily) [78] (Table 1). Trongtorsak et al. [78] found that LVEF and left ventricular end-systolic diameter were significantly improved by 2.9% (95% CI = 1.3%–4.5%, *p* = 0.001) and +2.1 mm (95% CI = 3.5–0.6 mm, *p* = 0.006), respectively; with no significant changes in left ventricular end-diastolic diameter (+1.0 mm, 95% CI = 3.74−1.82, *p* = 0.50, Table 1).

Conversely, a meta-analysis by Lei et al. [57], including nine trials and 2149 HF patients (CoQ10 supplementation 100 to 200 mg/d for 3–12 months, average seven months), reported no significant difference in LVEF between patients who received CoQ10 compared to placebo (+0.14; 95% CI = 0.08–0.37; *p* = 0.22, Table 1).

### 4.3. Effects of CoQ10 Supplementation on Functional Capacity

CoQ10 supplementation showed a significant increase in maximal exercise capacity [60] (Table 1). Although some authors reported no significant improvement in exercise duration or peak oxygen consumption (peak VO2) [62], Belardinelli et al. [63], in a small cohort of 23 patients (NYHA class II/III with stable HF of ischemic etiology supplemented with 100 mg orally of CoQ10 four times per day vs. placebo), reported a significant improvement in functional capacity (as measured by peak VO2) and in endothelial function (as expressed by endothelium-dependent dilation of the brachial artery). Notably, CoQ10 supplementation exerted a +9% increase in peak VO2, a +38% increase of endothelium-dependent dilation of the brachial artery and a significant decrease (−D12%) in systolic wall thickening score index [63]. Interestingly, exercise training (ET) exerted comparable effects to CoQ10 administration, thus reinforcing the need for performing training programs in HF patients [7,11,37,62,63,77,80,81,82]. CoQ10 supplementation induced a four-fold increase in baseline CoQ10 levels; notably, the combination strategy CoQ10 plus ET further increased levels with no reported side effects. In the randomized controlled trial conducted in 35 HF patients after three-month CoQ10 supplementation (150 mg/d), Rosenfeldt et al. [56] reported a significant increase in exercise capacity assessed by the Specific Activity Scale, treadmill exercise time and 6-min walking test distance (Table 1). A significant improvement of exercise capacity (measured as exercise duration, walking distance or both) have been reported in group undergoing CoQ10 supplementation (150 to 800 mg/d vs. placebo) in a recent meta-analysis including four clinical trial in 234 HF patients (SMD = 0.62; 95% CI = 0.02–1.12; *p* = 0.04) [57] (Table 1).

## 5. Effects of CoQ10 Supplementation on Mortality

Despite the association between worse clinical status and lower CoQ10 levels in HF patients, the prognostic role of CoQ10 levels is still debated. In 236 HF patients hospitalized for acute decompensating, increased CoQ10 levels improved survival (independent of clinical risk factors such as natriuretic peptides and renal function, hazard ratio (HR), 2.0; 95% CI = 91.2–3.3) [35]. However, in the CORONA trial (*n* = 1191), no association between CoQ10 levels and mortality has been reported [35] (Table 1)**.** Although CoQ10 levels reduction is induced by rosuvastatin use, no interaction between these two factors for any outcome was reported [50]. These results suggest that CoQ10 levels might be likely useful to stratify HF severity instead of the prognostic indicator.

In 1985, Langsjoen et al. [64] orally supplemented two groups of HF patients with NYHA class III or IV with CoQ10 versus a matched placebo. In this study, Group A patients received CoQ10 first and then placebo; conversely, Group B received placebo first and then CoQ10. Cardiac function indexes and CoQ10 levels were evaluated at 0 and 4 weeks (control stabilization period) and at 16 and 28 weeks (after the 12-week CoQ10/placebo-treatment periods). Group A showed significant improvement of cardiac function with an increase in CoQ10 levels during the CoQ10 supplementation phase which decreased during crossover to placebo. Specular findings were observed for Group B patients. All patients generally showed a brilliant clinical response to CoQ10 supplementation, with an increased survival in this cohort of patients usually experiencing a higher mortality rate at two years under conventional therapy [78] (Table 1).

The Q-SYMBIO trial [55] demonstrated a significant reduction of the two-year primary composite end-point of cardiovascular death, hospital admission for decompensating HF or need of mechanical support or cardiac transplant (30 vs. 57, *p* = 0.005; HR, 0.5; 95% CI = 0.32–0.80), death from cardiovascular causes (18 vs. 34, *p* = 0.039; HR, 0.51; 95% CI = 0.28–0.92) and all-cause mortality (21 vs. 39, *p* = 0.036; HR, 0.51; 95% CI = 0.30–0.89) in HF patients supplemented with CoQ10 (100 mg orally TID) versus placebo. The results of the Q-SYMBIO shed light on the potential beneficial effects of CoQ10 in HF; however, significant limitations should be acknowledged. First, the enrollment phase required a time period extension for up to eight years in more than 17 centers in nine different countries. These limitations could be ascribed to the researcher or site-related limitations, patient acceptance of drug administration, competing ongoing trials and other causes. The significant treatment effect consisting of about halved reduction in both primary composite end-point and all-cause mortality is quite unexpected and enthusiastic; however, it is plausible that either small number of events (mortality rate of 7% per year for the overall population) or the relatively small sample size might interfere with major findings; therefore results should be interpreted with caution. More recently, post-hoc analysis of baseline characteristics for short-term (three months) and long-term (two years) endpoints on the efficacy of CoQ10 in HF was investigated in a European cohort of 231 patients of the Q-SYMBIO trial (*n* = 420) [55]. After two years, all-cause mortality was lower in the CoQ10 group compared to placebo (10 (9%) vs. 24 (20%) patients, respectively), corresponding to a relative reduction of 53% (*p* = 0.04). Data also showed a significant reduction in hospitalization due to worsening HF in the CoQ10 group (3%) compared to placebo (13%, *p* = 0.007) [55].

Finally, Lei et al. meta-analyzed 14 trials (2149 HF patients, CoQ10 supplementation 100 to 200 mg/d for 3–12 months (average seven months)) showing a significant reduction in mortality among HF patients undergoing CoQ10 supplementation compared to placebo (RR = 0.69; 95% CI = 0.50–0.95; *p* = 0.02) [57] (Table 1).

## 6. CoQ10 Supplementation in HFpEF

Reduced ventricular compliance during the diastolic phase is a hallmark of clinically relevant HFpEF. In this case, the increase of left ventricular end-diastolic pressure occurs with normal left ventricular systolic function. Since the diastolic phase is sustained by ATP hydrolysis for disjoining myofilaments and consensual ventricular relaxation to occur, HFpEF can be viewed as energetic mismatch disorder [83]. In addition, inflammatory status and increased ROS production may lead to endothelial dysfunction driving to adverse cardiac remodeling and subsequent impaired ventricular relaxation [84,85,86,87] (Figure 1).

Based on pathophysiology studies and bench data on CoQ10’s role in mitochondrial energetics, the ability to attenuate inflammation and possibly improve endothelial function, investigating CoQ10 as a possible treatment strategy for HFpEF patients may represent an intriguing challenge. So far, CoQ10 supplementation (100 mg tid for 30 days) in HFpEF has been investigated in only one RCT; with no evidence of additional benefits on LV diastolic function in HFpEF patients with a short-term CoQ10 supplementation [73]. Although CoQ10 treatment group showed statistically significant improvement of average E/e’ 18.9 ± 3.8 vs. 15.1 ± 4.3; *p* < 0.01) and indexed left atrial volume (LAVI, 32 ± 9 mL/m^2^ vs. 26 ± 7 mL/m^2^; *p* < 0.05) together with the control group (18.4 ± 3.1) vs. 15.8 ± 5.6; *p* < 0.05) and (33 ± 7 mL/m^2^ vs. 30 ± 8 mL/m^2^; *p* < 0.05, respectively); there was no difference in change reduction between groups in ΔE/e’ and ΔLAVI (ΔE/e’ −3.6 vs. −2.4; *p* = 0.28 and ΔLAVI −5.4 vs. −4.4; *p* = 0.83, respectively) [73].

Analogously, only one trial investigated the effects of CoQ10 supplementation in patients with hypertrophic cardiomyopathy and diastolic dysfunction [64]. Compared to conventional therapy, patients with hypertrophic cardiomyopathy (*n* = 46) supplemented with CoQ10 showed a significant improvement in functional status (NYHA class ≥ 1), quality of life, 6-min walking test, and diastolic dysfunction (as evaluated by ≥1 parameter and in mitral regurgitation ≥1 grade). Post-treatment echocardiogram showed a significant reduction in the left ventricular outflow tract (LVOT) gradient of ≥15 mm Hg in obstructive cases (12 out of 46) in the treatment group. The mean interventricular septal thickness showed a 22.4% reduction (*p* < 0.005); and the mean posterior wall thickness showed a 23.1% reduction (*p* < 0.005) [64].

However, the small sample size strongly limits the conclusions in this cohort. Due to the evidence that current therapies may improve survival in patients with HFpEF, the research agenda should include trials testing the hypothesis that CoQ10 supplementation may improve survival.

## 7. Effects of CoQ10 Supplementation on Quality of Life (QoL)

In HF patients, common symptoms such as fatigue, exercise intolerance, inability in daily activities are linked to mitochondrial dysfunction and energy depletion. These symptoms have a strong impact on quality of life (QoL). In a double-blind placebo-controlled trial including 79 HF patients undergoing CoQ10 supplementation (100 mg/d for three months), Hofman-Bang et al. [59] reported a significant improvement in QoL score (Table 1). Conversely, Watson et al. [68] found no differences in the QoL score as evaluated by the Minnesota “Living with Heart Failure” questionnaire in the CoQ10 supplementation group (33 mg TID for three months) vs. placebo (Table 1).

## 8. CoQ10 Dosing, Duration of Supplementation and Drug Interactions in HF Trials

In all examined trials, CoQ10 doses ranging from 60 to 300 mg/d were orally administered. In 143 HF patients (NYHA class III/IV), CoQ10 supplementation (100 mg/d) resulted in an increase in CoQ10 levels from 0.85 to 2 mg/L associated to an increase in LVEF and to functional status improvement, with no reported adverse events [88].

Consequently, the highest CoQ10 level obtained in other studies was selected as the target in the Q-SYMBIO trial (2 mg/L), which used CoQ10 at 300 mg/d orally administered. Keogh et al. [52] reported a significant reduction in stroke index, pulmonary artery pressure and pulmonary capillary pressure in the cohort that reached CoQ10 levels of 3.25 ± 1.57 mg/L compared to placebo. Only one RCT has been conducted in patients with ischemic heart disease, which used CoQ10 at 300 mg/d for three months vs. placebo showing a significant reduction of inflammatory markers (i.e., tumor necrosis factor-α and interleukin-6) [89].

In a small sample size study (*n* = 7), Langsjoen et al. [90] focused on advanced HF patients (average LVEF = 22%), NYHA class IV, in which CoQ10 supplementation with CoQ10 (ubiquinone) 900 mg/d often did not attain therapeutic plasma CoQ10 levels (less than 2.5 mcg/mL); instead, therapeutic plasma levels (up to 6.5 mcg/mL) and significant improvement of EF (average up to 39%) and NYHA class (mean of IV to mean of II) were achieved supplementing these patients with reduced form of CoQ10 (ubiquinol) average 450 mg/d (therapeutic plasma level = >3.5 μg/mL).

Three months was the duration for the majority of examined trials, whereas the longest administration of CoQ10 was performed in the Q-SYMBIO trial (two years of CoQ10 supplementation) [61].

## 9. Conclusions

Overall, the relatively small sample size with sparse events reported, heterogeneous populations and study outcomes, different trial design and follow-up duration, different administered doses of CoQ10 and lack of use of novel HF drugs contribute to the uncertainty in evaluating and pooling data. Changes in the antioxidant systems in HF support the idea that CoQ10 may improve the outcome, quality of life and decrease morbidity and mortality. In recent years, the beneficial effects of CoQ10 supplementation in HF prevention and treatment have been consistently observed in many trials suggesting that CoQ10 may be considered as an adjunct to conventional treatment.

Several important issues on CoQ10 should be prioritized in the research agenda. The optimal dose of CoQ10 supplementation for HF patients should be defined. In this view, high-performance liquid chromatography may help to establish the plasma concentration, which is optimal for clinical effect. It also allows determining the normal levels of CoQ10, as well as adjusting the dose of administered CoQ10. The duration of CoQ10 supplementation in order to achieve clinical benefit in HF patients should be clearly defined. Better-powered studies are needed to assess the CoQ10 supplementation effect on survival in HF patients. Statins have been shown to decrease CoQ10 levels by inhibiting the mevalonate pathway. Statins can reduce blood serum levels of CoQ10 by almost much as 40%. It has been suggested that fatigue, muscle pain and weakness with statin use are related to a deficiency in CoQ10. Therefore, properly powered studies are needed in order to understand the role of CoQ10 supplementation in restoring CoQ10 levels in HF patients undergoing statin therapy. Furthermore, whether CoQ10 supplementation in HF on statin therapy may exert beneficial effects on the outcome by improving drug adherence remains to be elucidated.

## Figures and Tables

**Table 1:** Clinical Trials.

Trial	Population	Design	CoQ10 Dose and Duration	Results
Langsjoen et al. (1985) [64]	18 HF patients, NYHA class: III–IV	Double-blind and double-crossover trial	CoQ10 33 mg 3/d for 3 months	Significant improvement of SV and EF (*p* < 0.0001) measured by mean value from baseline to 12 weeks of CoQ10 supplementation vs. mean values after placebo period).
Permanetter et al. (1992) [65]	25 patients suffering from IDCM	Placebo-controlled, double-blind crossover trial	CoQ10 30 mg 4/d vs. placebo for 4 months	No statistically significant difference in ECG, LVEF (at rest and on exercise), LVESD, LVEDD, CI, SV, CT ratio, exercise tolerance and incidence of cardiac arrhythmias in chronic treatment with ubiquinone vs. placebo in patients suffering from IDCM.
Morisco et al. (1993) [52]	641HFrEF patients (mixed pathogeneses) NYHA class: III–IV	Double-blind placebo-controlled trial	CoQ10 50 mg 2/d or 3/d vs. placebo for 1 year	Decreased hospitalization for HF in CoQ10 group (*n* = 73) vs. placebo (*n* = 118; *p* < 0.001). Decreased episodes of pulmonary edema in CoQ10 group (*n* = 20) vs. placebo (*n* = 51; *p* < 0.001). Decreased episodes of cardiac asthma in CoQ10 group (*n* = 97) versus placebo (*n* = 198; *p* < 0.001).
Rengo et al. (1993) [66]	60 HFrEF patients NYHA class: III	Single-blind placebo randomized trial	CoQ10 100 mg/d vs. placebo for 7 months	Increase of LVEF % (15.79%) in CoQ10 group and decrease in placebo group (2%) vs. baseline (*p* < 0.001). Decrease of LVESD in CoQ10 (2%) and in placebo group (0.16%), (*p* < 0.001). Increase of FS in CoQ10 group (15.6%) vs. decrease in placebo group (2.19%, *p* < 0.001).
Baggio et al. (1994) [67]	2664 HF patients NYHA class: II and III	Open, non-comparative trial in 173 Italian centers	CoQ10 50–150 mg/d for 3 months	Significant improvement in BP, heart rate and respiratory rate, clinical signs and symptoms (at last 3 symptoms in 52.2% of patients) after CoQ10 supplementation.
Hofman-Bang et al. (1995) [59]	79 stable HFrEF patients	Double-blind, crossover placebo-controlled trial	CoQ10 50 mg 3/d vs. placebo for 3 months	CoQ10 therapy showed significant improvement in EF during a slight volume load: 25% ± 13% vs. 23% ± 12% (*p* < 0.05), EF at rest (mean value: 0.5, 95% CI = 1.0–2.0) increase of maximal exercise capacity: 100 ± 34 W vs. placebo 94 ± 31 W (*p* < 0.05), significantly increase of total score for the QoL assessment: 113 ± 22 vs. placebo 107 ± 23 (*p* < 0.05).
Watson et al. (1998) [68]	30 HFrEF patients (mixed pathogeneses)	Double-blind, crossover placebo-controlled trial	CoQ10 33 mg 3/d vs. placebo for 3 months	No significantly difference in EF from baseline (26% ± 6%) and after CoQ10 treatment (31% ± 9%) vs. placebo (*p* < 0.98), CI from baseline (2.7 ± 0.7) and after Coq10 treatment (2.9 ± 0.7) vs. placebo (*p* < 0.46), same results in Left Ventricular Diastolic Volume (*p* < 0.16) and Left Ventricular Systolic Volume (*p* < 0.26). No difference in QoL scores compared with placebo.
Munkholm et al. (1999) [60]	22 HF patients (mixed pathogeneses) NYHA class: II and III	Double-blind placebo-controlled randomized trial	CoQ10 100 mg 2/d vs. placebo for 1 year	Improvement of SI from baseline (31.28 ± 3.43) to 12 weeks in CoQ10 group (36.2 ± 2.72, *p* < 0.005) vs. no change in placebo group. Reduction of pulmonary capillary wedge pressure (PCWP) from baseline (40 ± 16 mm Hg) to 12 weeks in CoQ10 group (32 ± 15 mm Hg, *p* < 0.02) vs. no change in placebo group. Improvement in mean pulmonary artery pressure from baseline (27 ± 10 mm Hg) to 12 weeks in CoQ10 group (21 ± 7 mm Hg, *p* = 0.02) vs. no change in placebo group.
Khatta et al. (2000) [62]	55 HFrEF patients (mixed pathogeneses), NYHA class: III or IV	Randomized, double-blind placebo-controlled trial	CoQ10 200 mg/d vs. placebo for 6 months	No significantly difference in: Maximal oxygen consumption in CoQ10 group increase of 0.21 ± 3.4 mL/kg/min (95% CI = 1.25–1.68) vs. decrease in placebo group 0.49 ± 2.4 mL/kg/min (95% CI = 1.54–0.55) and in exercise duration 9.1 ± 3.4 min after CoQ10 treatment vs. 7.5 ± 2.9 min after placebo. No difference of EF measured by Radionuclide Ventriculography in CoQ10 group (decrease 0.3% ± 8%, CI = 3.7%–3.1%) vs. placebo (decrease 0.2% ± 8.6%, CI = 4.0%–3.6%).
Keogh et al. (2003) [52]	39 HFrEF patients (mixed pathogeneses) NYHA class: II or III	Randomized, double-blind placebo-controlled trial	CoQ10 50 mg 3/d vs. placebo for 3 months	Improvement of NYHA score in CoQ10 group (2.9 ± 0.06) from baseline (2.4 ± 0.12, *p* = 0.001) vs. no change between placebo and CoQ10 group (*p* = 0.01). No difference in CoQ10 group vs. placebo in Canadian-specific activity scale score (*p* = 0.29), 6 min walk test (*p* = 0.29) and Fractional Shortening (*p* = 0.9).
Berman et al. (2004) [53]	32 patients with HFrEF awaiting heart transplantation	Randomized controlled trial	CoQ10 60 mg 2/d vs. placebo for 3 months	Improvement in 6 min walk test from baseline (269.5–382.2 m, *p* < 0.0001) and vs. placebo group (254–177 m, *p* < 0.0001). Improvement of NYHA class in CoQ10 group from baseline (3.1–2.4, *p* = 0.01), no changes vs. placebo group (*p* = 0.01). No improvement in Fractional shortening.
Soongswang et al. (2005) [58]	15 idiopathic chronic DCM patients, median age: 4.4 years (range, 0.6–16.3)	Open-label prospective study	CoQ10 3.1 ± 0.6 mg/kg/d for 9 months	Significantly improvement of NYHA functional class (*p* < 0.005); CT Ratio: median 0.62 (0.55–0.78) vs. 0.58 (0.50–0.80, *p* < 0.022); QRS duration: median 82 msec (80–160) vs. 80 msec (60–149, *p* < 0.017), after supplementation therapy with CoQ10 when compared to baseline and post-discontinuation of CoQ10 at 9 months (depolarization in children with chronic idiopathic DCM).
Belardinelli et al. (2006) [63]	23 HF patients (secondary to ischemic heart disease) NYHA class II and III	Double-blind, placebo-controlled crossover trial	CoQ10 100 mg orally 4/d vs. placebo for 4 weeks	Significantly improvement of: peak VO_2_ after CoQ10 treatment (19.6 ± 4.8 mL/kg/min) and after CoQ10 + ET (21.5 ± 4.7ml/kg/min) vs. placebo (*p* < 0.0001); endothelium-dependent dilation of the brachial artery (EDDBA): 5.64% ± 1.95% compared with placebo: 4.19% ± 1.9% (*p* < 0.01); resting LVEF 43% ± 8.7% vs. placebo 37.9% ± 8% (*p* < 0.0023).
Kocharian et al. (2009) [69]	38 IDCM patients; < 18 years	Double-blind placebo-controlled trial	CoQ10 2 mg/kg/d over 2 or 3 doses increased to 10 mg/kg/d according to tolerance or side effects for 6 months	Improvement after CoQ10 supplementation therapy of CI (5.8 ± 4) vs. placebo (9 ± 4.2), *p* = 0.024; EF improvement: 42.1% ± 14.7% in CoQ10 group vs. placebo group 37.6% ± 9.7% (*p* < 0.267); FS improvement 18.5 ± 7.9 vs. placebo 14.9 ± 3.2 (*p* < 0.1).
Lee et al. (2013) [65]	51 Patients with 50% stenosis of one major coronary artery and treated with statin for last 1 month	Randomized placebo-controlled trial	CoQ10 300 mg/d for 12 weeks	Increase of plasma levels of CoQ10 (*P* < 0.001), antioxidant enzymes activities (*p* < 0.05) and vitamin E (*p* = 0.043) after CoQ10 supplementation vs. placebo. Decrease of inflammatory markers levels (TNF-α, *p* = 0.039) after CoQ10 supplementation.
Pourmoghaddas et al. (2014) [54]	62 patients with HFrEF (mixed pathogeneses) NYHA class: II–IV	Randomized double-blind placebo-controlled trial	CoQ10 100 mg 2/d with atorvastatin 10 mg/day vs. placebo for 4 months	Improvement of EF in CoQ10 group (24.2% ± 14.5%) from baseline 18.7 ± 10.3%, *p* = 0.003) and vs. placebo (26.2% ± 9.1% to 25.8% ± 9.7%, *p* = 0.006). Improvement of NYHA classification from baseline (2.7 ± 0.7) in CoQ10 group (2.3 ± 0.7, *p* = 0.025) and vs. placebo (2.9 ± 0.8 to 2.7 ± 0.7, *p* = 0.002).
Mortensen et al. (2014) [61]	420 HFrEF patients(mixed pathogeneses) NYHA class: I–II	Randomized double-blind placebo-controlled trial	CoQ10 100 mg orally 3/d vs. placebo for 2 years	Reduced risk of all-cause death in CoQ10 group: HR, 0.51 (95% CI = 0.30–0.89; *p* = 0.018); reduced composite risk including cardiovascular death, mechanical assist implantation, or urgent cardiac transplantation: HR, 0.50 (95% C =, 0.32–0.80; *p* = 0.003). No difference between groups for NYHA functional class, 6 min walk test or functional status.
Zhao et al. (2015) [70]	102 HF patients	Randomized double-blind, placebo-controlled trial	CoQ10 2 mg/kg/d divided in 2 or 3 doses for 1 year	Significant reduction in CoQ10 group of TNF-α, IL-6, hs-CRP and Malonylaldehyde plasma levels; significant increase of LVEF after 12 months (mean 46% ± 6%) vs. placebo (43% ± 5%, *p* < 0.05) and decrease of LVED (53 ± 3 mm) vs. placebo (54 ± 4 mm).
Chen et al. (2018) [71]	10 children diagnosed with DCM	Open-label trial	Liquid ubiquinol supplementation: 10 mg/kg body weight/d for 24 weeks	Liquid ubiquinol supplementation in children with DCM increased significantly the level of CoQ10 (3.9 ± 1.45 μM) from baseline (0.43 ± 0.12, *p* < 0.01). Improvement of EF after 24 weeks of treatment (65.67% ± 9.63%) from baseline (62.56 ± 7.47, *p* < 0.15) and FS after 24 weeks of treatment (36.59% ± 6.86%) from baseline (34.2 ± 5.22, *p* < 0.19). CoQ10 plasma level was moderately positively correlated with EF (*r* = 0.37, *p* = 0.28) and FS (*r* = 0.26, *p* = 0.45) after 24 weeks of supplementation.
Alehagen et al. (2018) [72]	443 elderly healthy participants	Prospective randomized double-blind placebo-controlled trial	CoQ10 200 mg/d for 4 years	Supplementation with CoQ10 and selenium for 4 years in elderly healthy subjects reduced significantly CV mortality (28.1%) vs. placebo (38.7%) after 12 years of follow-up; Reduced of CV mortality risk in treatment group in 12 years follow-up (HR: 0.58; 95% CI = 0.42–0.70; *p* = 0.007) significantly in patients with ischemic heart disease (HR: 0.52, *p* = 0.02, 95% CI = 0.3–0.9) diabetes (HR: 0.50, *p* = 0.03, 95% CI = 0.27–0.93), hypertension (HR: 0.59, *p* = 0.005, 95% CI = 0.41–0.85) and impaired functional capacity (NYHA III HR: 0.49, *p* = 0.02, 95% CI = 0.27–0.88).
Mortensen et al. (2019) [55]	420 HF patients (moderate to severe HF)	Randomized double-blind placebo-controlled trial	CoQ10 300 mg/d vs. placebo in addition to standard therapy for 2 years	Increase of CoQ10 plasma level (3.42 ± 0.21 μg/mL) from baseline (0.95 ± 0.08 μg/mL, *p* < 0.001) vs. decrease in placebo (0.76 ± 0.04 μg/mL). Reduction of NT-proBNP after 3 months of CoQ10 treatment vs. baseline (*p* = 0.052). Reduction of composite risk assessed by MACE: HR: 0.23; 95% CI = 0.11–0.51, *p* < 0.001. Improvement of at least 1 grade of NHYA class after 2 years of CoQ10 supplementation vs. placebo (48% vs. 25%, *p* = 0.003), significant improvement in Coq10 group of 6% from baseline in LVEF (*p* = 0.021) but not vs. placebo (*p* = 0.234).
Sobirin et al. (2019) [73]	30 HFpEF patients	Single center, unblinded randomized controlled trial	CoQ10 100 mg 3/d for 30 days	Decrease of E/e’ ratio in CoQ10 group (15.1 ± 4.3) vs. baseline (18.9 ± 3.8) and vs. placebo (15.8 ± 5.6); improvement in LAVI: 26 ± 7 mL/m^2^ vs. baseline 32 ± 9 mL/m^2^ (*p* = 0.04) and vs. placebo (30 ± 8 mL/m^2^); increase of LVEF: 56% ± 8% vs. baseline 55% ± 4% (*p* = 0.73) and vs. placebo (57% ± 7%).

**Captions:** AF: Atrial Fibrillation; CoQ10: Coenzyme Q10; CRP: C-reactive protein; CT ratio: Cardiothoracic ratio; CV mortality: Cardiovascular mortality; DCM: Dilated Cardiomyopathy; ECG: Electrocardiogram; EF: Ejection Fraction; ESR: erythrocyte sedimentation rate; ET: Exercise Training; FS: Fractional Shortening; HF: Heart Failure; HFpEF: Heart Failure with preserved Ejection Fraction; HFrEF: Heart Failure with reduced Ejection Fraction; HR: Hazard Ratio; hs-CRP: High Sensitivity C-Reactive Protein; IDCM: Idiopathic Dilated Cardiomyopathy; LAVI: Left Atrial Volume Index; LV: Left Ventricle; LVEF: Left Ventricular Ejection Fraction; LVESD: Left Ventricular End-Systolic Dimension; MACE: Major Adverse Cardiovascular Events; m: meters; msec: millisecond; NYHA: New York Heart Association; QoL: Quality of Life.

**Table 2:** Principal data from meta-analyses.

Meta-Analysis	Trial and Population	Coq10 Dose Range and Duration	Main Results
Soja et al. (1997) [74]	Number of included trials: 8 *n* = 356 patients with congestive HF	CoQ10 60–200 mg/d for average 7 months	Treatment with CoQ10 led to a statistically significant effect measured by SD: SV (0.71, *p* < 0.005), CO (0.61, *p* = 0.05), EF (1.37, *p* = 0.0001), CI (1.15, *p* = 0.0001) and EDVI (1.23, *p* = 0.0001).
Rosenfeldt et al. (2003) [56]	Number of included trials: 9 *n* = 824 patients with HF	CoQ10 90 to 100 mg/d for 4 to 8 weeks	CoQ10 supplementation showed increase of CoQ10 serum level (WMD 1.4, 95% CI = 1.3–1.5); improvement in EF at rest (1.9, 95% CI = 0.13–3.9), EF on exercise (−0.5, 95% CI = 3.9–2.9), maximum exercise capacity (14.2, 95% CI = –3.9–12.4), improvement in NYHA class score (−0.09, 95% CI = −0.037–0.18), improvement of exercise duration (1.0, 95% CI = −0.54–2.54) and reduction of mortality (OR: 0.76, 95% CI = 0.43–1.37).
Sander et al. (2006) [75]	Number of included trials: 11 *n* = 319 patients with HF	CoQ10 60 to 200 mg/d from 1 to 6 months	Significant improvement in EF (3.68%, 95% CI = 1.59–5.77), CI (0.32, 95% CI = −0.07–0.70), CO (0.28, 95% CI = 0.03–0.53), SI (5.80, 95% CI = 0.84–10.75) and SV (6.68, 95% CI = 20.41–13.78).
Fotino et al. (2013) [76]	Number of included trials: 13 *n* = 395 patients with CHF	CoQ10 60 to 300 mg/d, from 4 to 28 weeks	Supplementation with CoQ10 improved in HF patients: EF (3.67%, 95% CI = 1.60%–5.74%, 11 studies) and decreased NYHA score of 0.30 (95% CI = 0.66–0.06, 3 studies).
Madmani et al. (2014) [36]	Number of included trials: 7 *n* = 914 patients with CHF	CoQ10 vs. placebo, high-dose versus low-dose coenzyme Q10 for average 12 weeks	CoQ10 therapy increased plasma levels of CoQ10 (MD 1.46, 95% CI = 1.19–1.72, 3 studies); effects on LVEF (MD 2.26, 95% CI = 15.49–10.97, 2 studies) and on exercise capacity (12.79, 95% CI = 140.12–165.70, 2 studies) are unclear.
Yang et al. (2015) [77]	Number of included trials: 16 *n* = 1465 patients with CHF	CoQ10 100 mg to 450 mg/d for a period from 4 weeks to 2 years	CoQ10 supplementation therapy improved EF in 10 studies, improved exercise tolerance in 4 studies, reduced hospitalization and symptoms and improved survival (2 studies).
Trongtorsak et al. (2017) [78]	Number of included trials: 16 *n* = 1662 patients with CHF	CoQ10 100 mg/d to 200 mg/d	CoQ10 combined with standard therapy in HF improved CoQ10 level (MD 1.44 mcg/dL 95% CI = 1.16–1.73, *p* < 0.001). CoQ10 supplement improved LVEF (MD 2.9%, 95% CI = 1.3–4.5, *p* < 0.001) and LVESD (2.1 mm 95% CI = 3.5–0.6 mm, *p* < 0.006) but not LVEDD (1.0 mm 95% CI = 3.74–1.82, *p* < 0.50). All-cause death HR: 0.62 (95% CI = 0.40–0.95, *p* = 0.03), decrease of hospitalization vs. placebo HR: 0.39 (95% CI = 0.29 0.53, *p* < 0.001).
Lei et al. (2017) [57]	Number of included trials: 14 *n* = 2149 patients with CHF	CoQ10 100 mg/d to 200 mg/d for 3 to 12 months	CoQ10 supplementing therapy showed decrease of mortality vs. placebo: RR = 0.69, 95% CI = 0.50–0.95, *p* = 0.02, improvement of exercise capacity (exercise duration and/or walking distance) vs. placebo: SMD = 0.62, 95% CI = 0.02–0.30, (*p* = 0.04). No significant difference in LVEF vs. placebo: SMD 0.62, 95% CI = 0.02–1.12 (*p* = 0.04) and in NYHA score vs. placebo (SMD 0.70, 95% CI = 1.92–0.51 *p* = 0.26).
Flowers et al. (2014) [79]	Number of included trials: 6 *n* = 218 healthy adults or at high risk of CVD (without a diagnosis of CVD)	CoQ10 100 mg/d and 200 mg/d for 3 months	Unclear effects of CoQ10 supplementation on Diastolic BP (MD 1.62, 95% CI = −5.20–1.96)(2 studies), Total cholesterol (MD 0.30, 95% CI = 0.10–0.70, 1 study), HDL-cholesterol (MD 0.02, 95% CI = 0.13–0.17, 1 study), Triglycerides (MD 0.05, 95% CI = −0.42–0.52, 1 study).

**Captions:** CHF: Chronic Heart Failure; CI: Cardiac Index; CI: Confidence Intervals; CO: Cardiac Output; CoQ10: Coenzyme Q10; CVD: Cardiovascular Disease; EDVI: End-Diastolic Volume Index; EF: Ejection Fraction; HF: Heart Failure; HR: Hazard Ratio; LVEDD: Left Ventricular End-Diastolic Dimension; LVEF: Left Ventricular Ejection Fraction; LVESD: Left Ventricular End-Systolic Dimension; MD: Mean Difference; NYHA: New York Heart Association; OR: Odds Ratio, RR: Risk Ratio; SD: Standard Deviation; SI: Stroke Index; SMD: Standardized Mean Difference; SV: Stroke Volume; WMD: Weighted Mean Difference.
